# Supplementary material for: Predicting visual function by interpreting a neuronal wiring diagram
Source: Nature. 2024 Oct 2;634(8032):113–23. doi: 10.1038/s41586-024-07953-5 (PMC11446822; doi:10.1038/s41586-024-07953-5)
Supplement: Supplementary file 6 — CRF and ERF predictions for individual TmY4 and TmY9 cells. Analogous to Supplementary Data 3, but for TmY target types. Shown are the top four monosynaptic pathways, the strongest pathway passing through each of the top ten intermediary types (ranking from Extended Data Fig. 7), and the trisynaptic pathway Tm1–TmY–Dm3–TmY (see the section entitled Prediction of spatial normalization). [file 41586_2024_7953_MOESM6_ESM.zip › DataS4/TmY9q/720575940644567406.pdf]

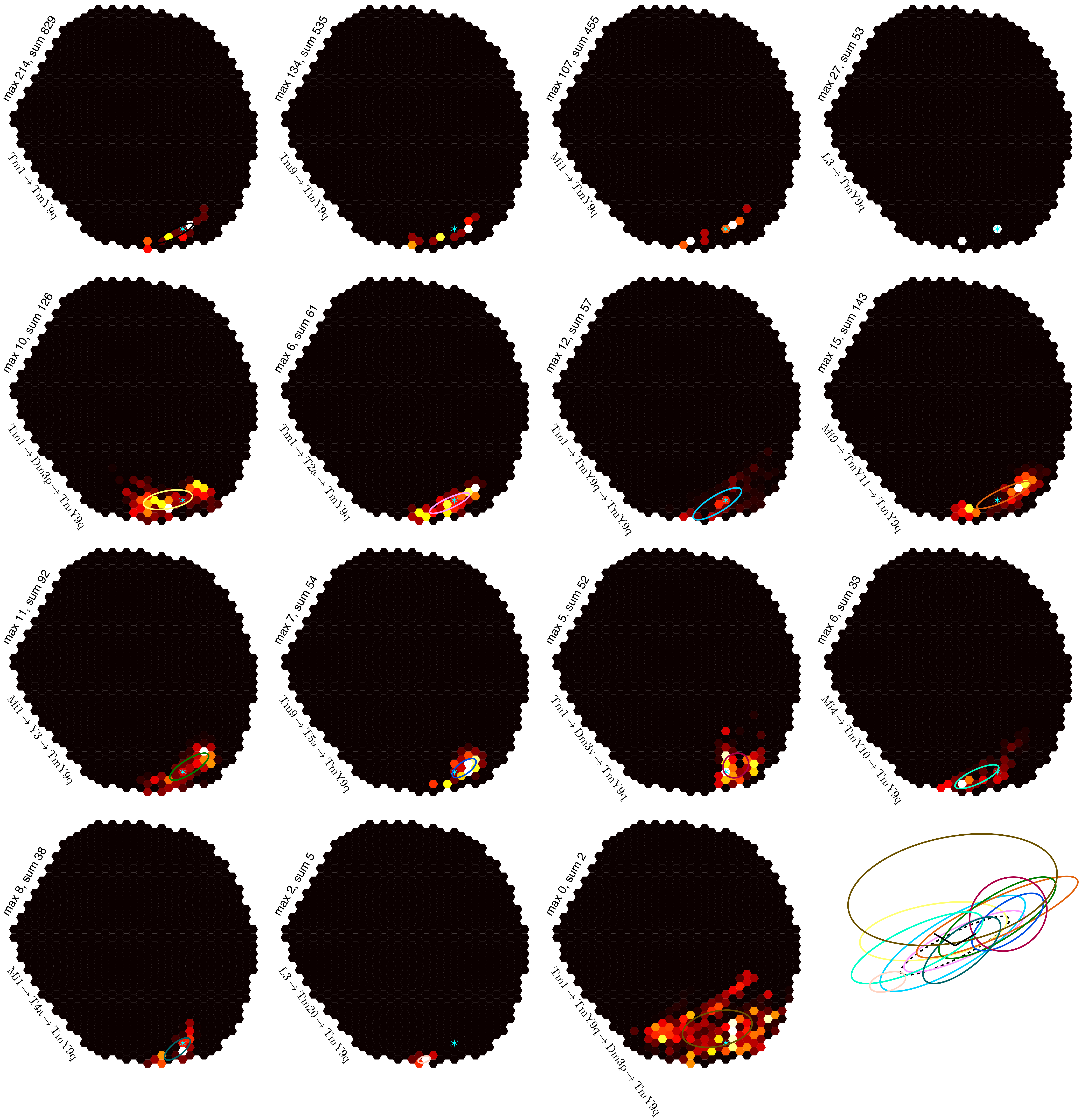
max 214, sum 829
Tm1 → TmY9q
max 134, sum 535
Tm9 → TmY9q
max 107, sum 455
Mi1 → TmY9q
max 27, sum 53
L3 → TmY9q
max 10, sum 126
Tm1 → Dm3p → TmY9q
max 6, sum 61
Tm1 → T2a → TmY9q
max 12, sum 57
Tm1 → TmY9q → TmY9q
max 15, sum 143
Mi9 → TmY11 → TmY9q
max 11, sum 92
Mi1 → Y3 → TmY9q
max 7, sum 54
Tm9 → T5a → TmY9q
max 5, sum 52
Tm1 → Dm3v → TmY9q
max 6, sum 33
Mi4 → TmY10 → TmY9q
max 8, sum 38
Mi1 → T4a → TmY9q
max 2, sum 5
L3 → Tm20 → TmY9q
max 0, sum 2
Tm1 → TmY9q → Dm3p → TmY9q
